# Association Between Osteoporosis and Adiposity Index Reveals Nonlinearity Among Postmenopausal Women and Linearity Among Men Aged over 50 Years

**DOI:** 10.1007/s44197-024-00275-9

**Published:** 2024-07-24

**Authors:** Po-Ju Chen, Yueh-Chien Lu, Sheng-Nan Lu, Fu-Wen Liang, Hung-Yi Chuang

**Affiliations:** 1 Department of Occupational Medicine, Kaohsiung Chang Gung Memorial Hospital, Kaohsiung, Taiwan; 2 Department of Family Medicine, Kaohsiung Chang Gung Memorial Hospital, Kaohsiung, Taiwan; 3 Chang Gung University College of Medicine, Taoyuan, Taiwan; 4 Division of Hepato-Gastroenterology, Department of Internal Medicine, Kaohsiung Chang Gung Memorial Hospital, Kaohsiung, Taiwan; 5 Department of Public Health, College of Health Sciences, Kaohsiung Medical University, Kaohsiung, Taiwan; 6 Department of Medical Research, Kaohsiung Medical University Hospital, Kaohsiung Medical University, Kaohsiung, Taiwan; 7 Center for Big Data Research, Kaohsiung Medical University, Kaohsiung, Taiwan; 8 Department of Community Medicine, Kaohsiung Medical University Hospital, No. 100, Shih-Chuan 1st Road, Sanmin Dist., Kaohsiung, 80708 Taiwan; 9 Ph.D. Program in Environmental and Occupational Medicine, College of Medicine, and Research Center for Precision Environmental Medicine, Kaohsiung Medical University, Kaohsiung, Taiwan

**Keywords:** Obesity, Osteoporosis, Abdominal volume index, Visceral adiposity index, Generalized additive model, Segmented regression model

## Abstract

**Purpose:**

Previous research shows conflicting views on the relationship between obesity and osteoporosis, partly due to variations in obesity classification and the nonlinear nature of these relationships. This study investigated the association between adiposity indices and osteoporosis, diagnosed using dual-energy X-ray absorptiometry (DXA), employing nonlinear models and offering optimal thresholds to prevent further bone mineral density decline.

**Methods:**

In 2019, a prospective study enrolled males over 50 years and postmenopausal women. Anthropometric measurements, blood biochemistry, and osteoporosis measured by DXA were collected. Associations between adiposity indices and osteoporosis were analyzed using a generalized additive model and segmented regression model.

**Results:**

The study included 872 women and 1321 men. Indices such as abdominal volume index (AVI), visceral adiposity index (VAI), waist circumference (WC), hip circumference, body mass index (BMI), waist-to-hip ratio, and waist-to-height ratio (WHtR) were inversely associated with osteoporosis. In women, the relationship between the risk of osteoporosis and the adiposity indices was U-shaped, with thresholds of WC = 94 cm, AVI = 17.67 cm^2^, BMI = 25.74 kg/m^2^, VAI = 4.29, and WHtR = 0.61, considering changes in bone mineral density. Conversely, men exhibited a linear patterns for the inverse association.

**Conclusion:**

The impact of obesity and adiposity on osteoporosis varies significantly between women and men. In postmenopausal women, the relationship is nonlinear (U-shaped), with both very low and very high adiposity linked to higher osteoporosis risk. In men over 50, the relationship is linear, with higher adiposity associated with lower osteoporosis risk. The study suggests that maintaining specific levels of adiposity could help prevent osteoporosis in postmenopausal women.

## Introduction

Osteoporosis, characterized by reduced bone mineral density (BMD) and microarchitectural deterioration, poses a significant health risk, particularly in older individuals. Fragility fractures, particularly of the hips or vertebrae, are common comorbidities. An estimated 2.6 million hip fractures are anticipated globally by 2025, with projections indicating a potential increase to 7.3–21.3 million [1]. The annual worldwide cost of osteoporosis is estimated at $25.3 billion USD worldwide [2].

The global prevalence of obesity increased to 36.9% in males and 38.0% in females from 1980 to 2013 [3]. Estimates suggest that obesity may affect 51% of the total population [4], with an anticipated worldwide burden of approximately 573 million by 2030 [5]. Although obesity is widely accepted to be associated with increased cardiovascular events and all-cause mortality, some paradoxical findings have emerged. Early studies have indicated that overweight or obese patients on dialysis exhibit a lower mortality risk in a dialysis patient cohort [6, 7], indicating potential benefits of being slightly overweight in certain circumstances. Additionally, nonlinear U-shaped associations have been observed between obesity and all-cause mortality in patients with type 2 diabetes [8]. Aune et al. [9] found a J-shaped association between body mass index (BMI) and mortality in a cohort of healthy individuals, with the lowest risk observed at a BMI of 22–23 kg/m^2^. Therefore, identifying the ideal body weight with the least health consequences would be crucial. The U-shaped or J-shaped nature of this relationship may help clarify findings when different segments of the results were examined in isolation. Similarly, the importance of the nonlinear relationship between adiposity and osteoporosis warrants further study.

The association between obesity and osteoporosis remains controversial due to the intricate interactions, including direct mechanical loads and cellular effects, between adipocyte and osteoblasts. Traditionally, body weight has been considered beneficial for bone formation, relying on the adaptive response of bone cells to mechanical loading, which enhances metabolism, increases growth factor production, and synthesizes the bone matrix [10]. This suggests that weight can prevent bone loss and osteoporosis. Prior obesity estimations relied on indices such as body weight (BW) or BMI, although these measures do not adequately reflect obesity, especially because they provide limited information about body composition [11]. Hence, several indices have been developed that emphasize adiposity and target abdominal obesity, such as the waist-to-hip ratio (WHR), waist-to-height ratio (WHtR), abdominal volume index (AVI), and visceral adiposity index (VAI). The development of new indices that consider adipose tissue, rather than solely height and body weight, provides a better understanding of an individual’s metabolic state. Scott et al. have highlighted the importance of higher adiposity in increasing the likelihood of sarcopenia and osteoporosis [12]. Therefore, evaluating the association between osteoporosis and indices considering adipose tissue, such as AVI and VAI, may be more reliable than traditional indices. However, the effect of adiposity varies across different subject groups. Studies by Jankowska et al., which recruited males aged 20–60, demonstrated a decreased in bone mass with increased adiposity assessed by WHR [13]. In contrast, Chang et al. specified an increased osteoporotic risk with low central obesity, evaluated using WC, in women aged above 65 [14]. These inconsistent findings emphasize the need for further evaluations of adiposity effects on osteoporosis.

Given that studies suggest a U-shaped or J-shaped relationship between BMI and health outcomes [8, 9], it is essential to recognize the nonlinear nature of adiposity and its association with health, which necessitates further investigation into effects on osteoporosis. Several mechanisms have been proposed in relation to fat and bone metabolism. The complexity of this interaction includes the secretion of biologically active molecules, such as adiponectin, estrogen, interleukin-6, from adipocytes [15]. Additionally, adipocytes and bone cells share the same progenitor cells, the mesenchymal stem cells, which participate in the homeostasis of both cell types [15]. The phenomenon suggests a continuous interaction, prompting our study design to explore a nonlinear model between adipose tissue and osteoporosis. Our hypothesis posits a continuous interaction between adiposity and bone mineral density in both postmenopausal women and men over the age of 50, considering the nonlinear nature of this relationship. The goal of this study is to investigate the association between various adiposity indices and osteoporosis diagnosed using dual-energy X-ray absorptiometry (DXA), employing nonlinear models.

## Methods

### Study Population

In this prospective study conducted between January 1, 2019, and December 31, 2019, data were collected from 3,778 participants. The participants in the current study were recruited during voluntary self-payment or regular health examinations, which included dual-energy X-ray absorptiometry (DXA) scans to assess BMD. The majority of participants did not exhibit symptoms and were free of osteoporotic fracture at the time of recruitment. Men aged under 50 years, pre-menopausal women those with missing anthropometric or biomedical measurements, and participants undergoing medical treatment for osteoporosis such as bisphosphonates, nuclear factor-κB ligand inhibitors, and selective estrogen receptor modulators, were excluded. This left 2193 participants, including 872 women and 1321 men, for the final analysis.

This study was approved by the Institutional Review Board of the Chang Gung Medical Foundation (No. 202000576B0C601). All participants provided informed written consent before the study.

BMD (g/cm^2^) was measured using a DXA scanner (Hologic Corp., Waltham, MA, USA) at the lumbar vertebrae (L1–L4), femoral neck, and total hip. Daily quality control was performed according to the service manual from the manufacturer, Hologic Corporation, using the software and quality control phantom to ensure the system was function correctly. T-scores were established by the World Health Organization (WHO) as the number of standard deviations of an individual’s BMD from a database of U.S. Caucasian women aged 20–29 years, based on the Third National Health and Nutrition Examination Survey [16]. T-scores ≥ -1.0 were classified as normal, those ranging from − 1.0 to − 2.5 were considered indicative of low bone mass, and scores ≤ -2.5 were diagnosed as osteoporosis. Participants with the lowest T-score ≤ -2.5 among the lumbar spine, femoral neck or total hip were diagnosed with osteoporosis based on the consensus guidelines from the American Association of Clinical Endocrinologists (AACE) [17].

### Anthropometric and Adiposity Indices

Basic information and anthropometric data, including sex, age, tobacco use, alcohol consumption, betel nut habits, and menopausal status, were collected from questionnaires and interviews conducted by trained nurses. WC and HC were measured following WHO protocol, with participants standing with their arms relaxed at their sides, feet together, and weight evenly balanced on both feet [18]. Serum levels of fasting plasma glucose, blood urea nitrogen (BUN), alanine aminotransferase (ALT), aspartate aminotransferase, gamma-glutamyl transferase (GGT), total cholesterol, high-density lipoprotein (HDL), low-density lipoprotein (LDL), triglycerides (TG), calcium and albumin were assessed. The clinical biochemical assessments were conducted in the central laboratory at Kaohsiung Chang Gung Memorial Hospital, which is accredited as a medical center and university hospital. The laboratory is also accredited by the College of American Pathologists (CAP). Traditional adiposity indices, including WC, BMI, WHR, and WHtR, were examined alongside novel indices such as AVI [19] and VAI [20]. AVI estimates abdominal volume by conceptualizing the body as a cylinder or vertical cone [19]. It correlates significantly with visceral fat [21] and provides insights into metabolic health, serving as a predictor for metabolic syndrome and being associated with insulin resistance [22]. VAI assesses visceral fat using WC, BMI, HDL, and TG, with sex-specific risk estimation [20]. It also correlates significantly with visceral fat [23] and contributes to predicting metabolic syndrome [24] and insulin resistance [25]. Novel adiposity indices were calculated using the following formulas:

1 $$ {\text{AVI = }}\frac{{\left\{ {{2} \times {\text{WC}}^2 {\text{(cm)}} + {0}{\text{.7}} \times \left[ {{\text{WC}}({\text{cm}}) - {\text{HC}}({\text{cm}})} \right]^2 } \right\}}}{{{1000}}} $$

2 $$ {\text{VAI}}_{{\text{female}}} = \left[ {\frac{{{\text{WC}}\;{\text{(cm)}}}}{{{36}{\text{.58}} + \left( {{1}{\text{.89}} \times {\text{BMI}}} \right)}}} \right] \times \left[ {\frac{{{\text{TG}}\;{\text{(mmol/l)}}}}{{{0}{\text{.81}}}}} \right] \times \left[ {\frac{{{1}{\text{.52}}}}{{{\text{HDL}}({\text{mmol}}/{\text{l}})}}} \right] $$

3 $$ {\text{VAI}}_{{\text{male}}} = \left[ {\frac{{\text{WC(cm)}}}{{{39}{\text{.68}} + \left( {{1}{\text{.88}} \times {\text{BMI}}} \right)}}} \right] \times \left[ {\frac{{\text{TG(mmol/l)}}}{{{1}{\text{.03}}}}} \right] \times \left[ {\frac{{{1}{\text{.31}}}}{{\text{HDL(mmol/l)}}}} \right] $$

Traditional adiposity indices also contribute to assessing to body fat [26] and visceral fat [27], computed using the formulas provided below:

4 $$ {\text{BMI}} = \frac{{\text{BW(kg)}}}{{{\text{BH}}^2 {\text{(m)}}}} $$

5 $$ {\text{WHR}} = \frac{{\text{WC(cm)}}}{{\text{HC(cm)}}} $$

6 $$ {\text{WHtR}} = \frac{{\text{WC(cm)}}}{{\text{BH(cm)}}} $$

### Statistical Analysis

Descriptive statistics were used to summarize the mean and standard deviation for continuous variables, and frequencies and percentages for categorical variables. Between-group comparisons were performed using the chi-square test or Fisher’s exact test for categorical variables and Student’s *t*-test for continuous variables. Fisher’s exact test was specifically applied when the expected frequencies in the contingency table were less than 5. Pearson’s correlation coefficient was employed to identify the relationships among various adiposity indices. A univariable logistic regression was used to examine the association between the adiposity indices and osteoporosis. Variables that reached significance (*p* < 0.05) were incorporated into the multivariable logistic regression analysis, excluding those with potential collinearity. Specifically, variables with a Pearson’s correlation coefficient above 0.7 were removed from the analysis to prevent collinearity. Multiple logistic regressions were applied to control for confounders, including age, high-density lipoprotein, low-density lipoprotein, triglycerides, uric acid, calcium, albumin, smoking, and alcohol consumption. The selection of potential confounders was based on their established associations with both the exposure and osteoporosis in the existing literature [14, 28, 29]. Since VAI involves TG and HDL in its calculation, the confounders selected for regressions were adjusted solely for age, low-density lipoprotein, uric acid, calcium, albumin, smoking, and alcohol consumption. A *p*-value < 0.05 was considered statistically significant. Odds ratios with corresponding 95% confidence intervals (CIs) were presented, and multiple logistic regression models were used to generate receiver operating characteristic (ROC) curves, including calculation of the area under the curve (AUC). Statistical analyses were conducted using SPSS software (version 20.0; IBM Corp., Armonk, NY, USA) for data description.

Generalized Additive Models (GAMs) offer an advantage in their ability to avoid assuming specific functional forms. These models utilize smoothing techniques such as splines to fit curves to the data, thereby improving pattern capture while minimizing noise. Consequently, GAMs were used to identify nonlinear relationships between the variables and the binary outcome of osteoporosis. The GAM integrates variables additively through a link function to establish connections between smoothed and response variables. The typical structure of a GAM is defined as

7 $$ g(E(y)) = \beta_0 + s(x_1 ) + s(x_2 ) + \cdots + s(x_n ) $$

where $s(x_j )$ is a smooth function of the variable $x_j$, and g and y are the link function and response variable, respectively. The effective degree of freedom (EDF) value distinguished linear or nonlinear relationships with an EDF of 1 indicating a linear association between explanatory variables and response variable. In this study, the default EDF settings were used. GAM with a logit link function and restricted maximum likelihood criteria for smoothness selection of each explanatory variable were adopted to assess the associations between osteoporosis and adiposity indices, adjusting for age, HDL, LDL, TG, uric acid, Ca, albumin, smoking, and alcohol consumption. Slope changes were expected between the dependent and independent variables based on GAM; therefore, segmented regression models (SRM) were applied. Segmented regression, also known as broken-line or piecewise regression, is a statistical method that assumes that the variables have different linear relationships conjoined at certain points. These points are regarded as breakpoints, and can be interpreted as thresholds for understanding. The advantage of SRM lies in their ability to capture and quantify changes in the relationship between segments, allowing for the identification of breakpoints where variables changes significantly. The model assumes linearity within each segment and continuity at breakpoints. The Davies test was used to determine for the existence of a change point within the range of data. Using the SRM, we identified the potential threshold for each adiposity index in relation to bone mineral density. The R 4.2.0 software, along with the mgcv and segmented packages, were utilized to implement the GAM and segmented regression [30, 31].

## Results

### Demographic Characteristics

In this study, 2193 participants were included; 559 diagnosed with osteoporosis and 1634 without osteoporosis. Table 1 presents the anthropometric characteristics of all participants stratified by sex. There were 872 postmenopausal women and 1321 men aged > 50 years. Patients with osteoporosis were older than those without osteoporosis, and non-osteoporotic participants exhibited higher values of traditional anthropometric measurements, including WC, HC, WHR, WHtR, BH, BW, and BMI. Novel anthropometric indicators, such as AVI and VAI, followed trends similar to those of traditional indicators; however, VAI did not demonstrate statistical significance.

**Table 1:** Demographic characteristics of all participants and subgroups

	Total (n = 2193)			Females (n = 872)			Males (n = 1321)		
	Osteoporosis^a^ (n = 559)	Non-osteoporosis (n = 1634)	*p* value	Osteoporosis^a^ (n = 346)	Non-osteoporosis (n = 526)	*p* value	Osteoporosis^a^ (n = 213)	Non-osteoporosis (n = 1108)	*p* value
Age (years)	64.22 ± 7.40	60.55 ± 6.77	< 0.001*	64.51 ± 7.53	59.17 ± 6.45	< 0.001*	63.74 ± 7.18	61.20 ± 6.82	< 0.001*
BMD (g/cm^2^)	0.71 ± 0.09	0.90 ± 0.11	< 0.001*	0.70 ± 0.09	0.85 ± 0.10	0.009*	0.73 ± 0.07	0.92 ± 0.11	< 0.001*
WC (cm)	78.52 ± 9.99	84.66 ± 9.83	< 0.001*	75.89 ± 9.91	78.57 ± 9.30	< 0.001*	82.80 ± 8.54	87.55 ± 8.69	< 0.001*
HC (cm)	90.99 ± 6.03	94.18 ± 6.32	< 0.001*	91.30 ± 6.37	94.00 ± 7.22	< 0.001*	90.49 ± 5.41	94.26 ± 5.85	< 0.001*
WHR	0.86 ± 0.08	0.90 ± 0.07	< 0.001*	0.83 ± 0.07	0.84 ± 0.06	0.293	0.91 ± 0.06	0.93 ± 0.06	0.002*
WHtR	0.49 ± 0.06	0.51 ± 0.06	< 0.001*	0.49 ± 0.07	0.50 ± 0.06	0.088	0.50 ± 0.05	0.52 ± 0.05	< 0.001*
BH (cm)	158.99 ± 7.90	165.04 ± 7.55	< 0.001*	154.70 ± 5.25	157.67 ± 5.21	< 0.001*	165.95 ± 6.39	168.54 ± 5.76	< 0.001*
BW (kg)	59.08 ± 10.26	69.19 ± 11.45	< 0.001*	55.26 ± 8.79	69.83 ± 9.40	< 0.001*	65.30 ± 9.43	73.16 ± 10.11	< 0.001*
BMI (kg/m^2^)	23.33 ± 3.43	25.33 ± 3.45	< 0.001*	23.11 ± 3.67	24.48 ± 3.69	< 0.001*	23.68 ± 2.98	25.74 ± 3.25	< 0.001*
AVI	12.68 ± 3.13	14.62 ± 3.33	< 0.001*	11.91 ± 3.08	12.71 ± 2.98	< 0.001*	13.92 ± 2.80	15.53 ± 3.10	< 0.001*
VAI	1.64 ± 3.26	1.87 ± 1.62	0.323	1.50 ± 1.28	1.78 ± 1.41	0.049*	1.86 ± 5.01	1.91 ± 1.71	0.525
Current smoker, n (%)	67 (12.0%)	226 (13.8%)	0.268	4 (1.2%)	9 (1.7%)	0.508	63 (29.6%)	217 (19.6%)	0.001*
Betelnut, n (%)	22 (3.9%)	81 (5.0%)	0.324	2 (0.6%)	3 (0.6%)	> 0.999^b^	20 (9.4%)	78 (7.0%)	0.231
Alcohol, n (%)	113 (20.2%)	488 (29.9%)	< 0.001*	48 (13.9%)	110 (20.9%)	0.008*	65 (30.5%)	378 (34.1%)	0.308

BMD, bone mineral density; WC, waist circumference; HC, hip circumference; WHR, waist-to-hip ratio; WHtR, waist-to-height ratio; BH, body height; BW, body weight; BMI, body mass index; AVI, abdominal volume index; VAI, visceral adiposity index

*Statistical significance with a *p* value < 0.05

^a^Osteoporosis was diagnosed when the lowest T-score among the lumbar spine, femoral neck or total hip is ≤ -2.5

^b^Fisher’s exact test

Table 2 presents the biochemical profiles of all the participants categorized by sex, with statistical differences determined using Student’s t-test. Participants with osteoporosis had significantly higher total cholesterol and HDL levels, whereas participants without osteoporosis had higher diastolic blood pressure, creatinine, uric acid, ALT, triglyceride, and albumin levels. In women with osteoporosis, BUN and HDL levels significantly increased, whereas uric acid and triglyceride levels decreased. Male patients with osteoporosis exhibited significantly higher levels of GGT and HDL, whereas their calcium and albumin levels were significantly lower. However, the differences between osteoporotic and non-osteoporotic groups were minor and statistically significant, but did not necessitate changes in clinical practice.

**Table 2:** Biochemistry characteristics of all participants and subgroups

	Total (n = 2193)			Females (n = 872)			Males (n = 1321)		
	Osteoporosis^a^ (n = 559)	Non-osteoporosis (n = 1634)	*p* value	Osteoporosis^a^ (n = 346)	Non-osteoporosis (n = 526)	*p* value	Osteoporosis^a^ (n = 213)	Non-osteoporosis (n = 1108)	*p* value
SBP (mmHg)	127.48 ± 21.80	127.50 ± 19.98	0.981	126.90 ± 22.72	125.58 ± 21.17	0.380	128.41 ± 20.25	128.42 ± 19.34	0.997
DBP (mmHg)	81.98 ± 10.99	84.58 ± 10.91	< 0.001*	79.49 ± 10.90	79.81 ± 11.25	0.672	86.02 ± 9.90	86.84 ± 9.98	0.271
FPG (mg/dL)	102.40 ± 24.04	105.34 ± 26.95	0.022*	102.23 ± 22.84	103.65 ± 26.94	0.420	102.66 ± 25.93	106.14 ± 26.93	0.083
HbA1c (%)	5.94 ± 0.93	6.01 ± 0.96	0.113	5.93 ± 0.89	5.93 ± 0.87	0.987	5.94 ± 0.99	6.05 ± 1.00	0.154
BUN (mg/dL)	11.62 ± 4.65	11.89 ± 5.32	0.288	11.36 ± 5.02	10.20 ± 3.64	< 0.001*	12.03 ± 3.98	12.68 ± 5.78	0.119
Creatinine (mg/dL)	0.81 ± 0.38	0.90 ± 0.46	< 0.001*	0.73 ± 0.43	0.69 ± 0.12	0.157	0.95 ± 0.21	1.00 ± 0.52	0.150
Uric acid (mg/dL)	5.71 ± 1.44	6.24 ± 1.46	< 0.001*	5.25 ± 1.18	5.47 ± 1.36	0.012*	6.45 ± 1.51	6.60 ± 1.36	0.142
ALT (U/L)	25.35 ± 24.94	28.18 ± 22.03	0.011*	22.86 ± 15.49	24.23 ± 19.70	0.274	29.39 ± 34.93	30.01 ± 22.83	0.726
AST (U/L)	26.79 ± 15.71	27.26 ± 16.75	0.560	25.42 ± 10.31	26.13 ± 18.07	0.508	29.02 ± 21.64	27.80 ± 16.07	0.341
GGT (U/L)	17.88 ± 40.98	18.96 ± 27.66	0.497	12.36 ± 10.44	14.68 ± 31.63	0.197	27.02 ± 64.50	21.01 ± 25.30	0.026*
Total cholesterol (mg/dL)	208.70 ± 40.07	202.25 ± 40.85	0.001*	214.28 ± 39.86	212.53 ± 39.97	0.527	199.64 ± 38.82	197.46 ± 40.38	0.451
HDL (mg/dL)	54.59 ± 15.10	49.18 ± 12.85	< 0.001*	58.71 ± 15.20	55.83 ± 13.07	0.004*	47.92 ± 12.33	46.03 ± 11.48	0.030*
LDL (mg/dL)	126.97 ± 34.55	124.10 ± 35.16	0.094	129.26 ± 35.20	129.21 ± 35.27	0.983	123.26 ± 33.22	121.68 ± 34.86	0.540
Triglycerides (mg/dL)	109.86 ± 105.42	131.59 ± 91.81	< 0.001*	97.21 ± 55.77	112.43 ± 63.63	< 0.001*	130.34 ± 153.20	140.65 ± 101.25	0.216
Ca (mg/dL)	9.51 ± 0.39	9.50 ± 0.34	0.783	9.56 ± 0.41	9.56 ± 0.35	0.915	9.43 ± 0.34	9.48 ± 0.34	0.046*
Albumin (g/dL)	4.63 ± 0.26	4.67 ± 0.24	0.001*	4.63 ± 0.25	4.65 ± 0.2	0.164	4.63 ± 0.27	4.67 ± 0.24	0.011*

SBP, systolic blood pressure; DBP, diastolic blood pressure; FPG, fasting plasma glucose; HbA1c, hemoglobin A1C; BUN, blood urea nitrogen; ALT, alanine aminotransferase; AST, aspartate aminotransferase; GGT, gamma-glutamyltransferase; HDL, high-density lipoprotein; LDL, low-density lipoprotein; Ca, calcium

*Statistical significance with a *p* value < 0.05

^a^Osteoporosis was diagnosed when the lowest T-score among the lumbar spine, femoral neck or total hip is ≤ -2.5

### Linear Models of Adiposity Indices

Before the multiple logistic regression analysis, collinearity was assessed using the Pearson’s correlation coefficient (*r*), as shown in Table 3. The correlation patterns between the indices were similar for both sexes. Among postmenopausal women, the novel indicator AVI was strongly correlated with various indices, including BW (*r* = 0.83), BMI (*r* = 0.86), WC (*r* = 0.99), HC (*r* = 0.79), WHR (*r* = 0.76), and WHtR (*r* = 0.96). In the men’s group, AVI was highly correlated with BW (*r* = 0.85), BMI (*r* = 0.87), WC (*r* = 0.996), HC (*r* = 0.81), WHR (*r* = 0.78), and WHtR (*r* = 0.94). However, the VAI did not exhibit a strong relationship with other indices for women and men.

**Table 3:** Pearson’s correlation coefficients between anthropometric indices

	Postmenopausal women								
	BH	BW	BMI	WC	HC	WHR	AVI	WHtR	VAI
BH	1	0.32*	-0.11*	0.02	0.17*	-0.13*	0.02	-0.26*	0.03
BW		1	0.90*	0.82*	0.88*	0.43*	0.83*	0.71*	0.29*
BMI			1	0.85*	0.85*	0.50*	0.86*	0.86*	0.28*
WC				1	0.77*	0.80*	0.99*	0.96*	0.36*
HC					1	0.23*	0.79*	0.70*	0.18*
WHR						1	0.76*	0.80*	0.39*
AVI							1	0.96*	0.35*
WHtR								1	0.34*
VAI									1

	Men over 50 years old								
	BH	BW	BMI	WC	HC	WHR	AVI	WHtR	VAI
BH	1	0.46*	-0.03	0.15*	0.33*	-0.09*	0.15*	-0.19*	0.02
BW		1	0.87*	0.84*	0.88*	0.46*	0.85*	0.68*	0.17*
BMI			1	0.87*	0.82*	0.56*	0.87*	0.87*	0.17*
WC				1	0.80*	0.79*	**0.99**6*	0.94*	0.20*
HC					1	0.27*	0.81*	0.68*	0.11*
WHR						1	0.78*	0.81*	0.21*
AVI							1	0.94*	0.19*
WHtR								1	0.19*
VAI									1

BH, body height; BW, body weight; BMI, body mass index; WC, waist circumference; HC, hip circumference; WHR, waist-to-hip ratio; AVI, abdominal volume index; WHtR, waist-to-height ratio; VAI, visceral adiposity index

*Statistical significance with a *p* value < 0.05

^a^A Pearson’s correlation coefficient (r) above 0 indicates a positive relationship, whereas a coefficient below 0 indicates a negative relationship. A strong correlation is identified when 0.7 ≤|r|< 1

**Fig. 1:**
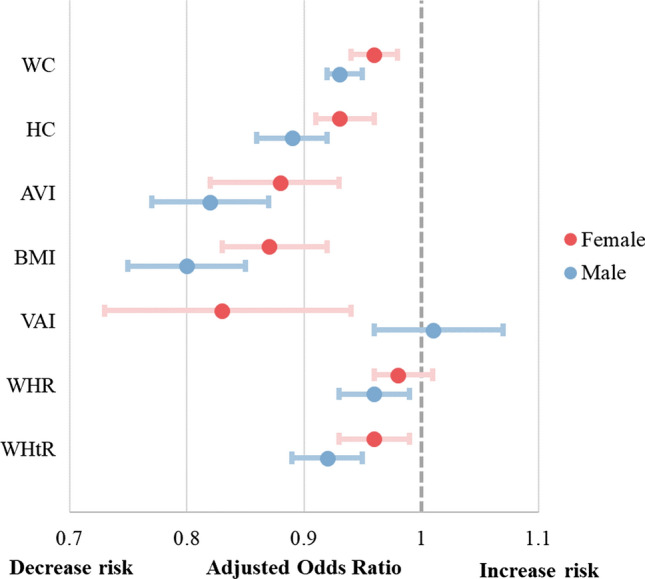
Adjusted odds ratio between adiposity indices and risk of osteoporosis in both genders. WC, waist circumference; HC, hip circumference; AVI, abdominal volume index; BMI, body mass index; VAI, visceral adiposity index; WHR, waist-to-hip ratio; WHtR, waist-to-height ratio

Table 4 and Fig. 1 presents the results of the multivariable logistic regression, which incorporates significant variables identified in simple logistic regression while excluding those exhibiting collinearity based on Pearson’s correlation. This approach aims to enhance the precision and reliability of ORs. Most indices exhibited protective effects (OR < 1) and remained statistically significant in both crude and adjusted models, implying robust and possibly independent associations with osteoporosis**.** Conversely, neither WHR in women nor VAI in men showed significance in either model. Notably, WHtR in postmenopausal women achieved significance after adjusting for confounding variables, suggesting its potential relevance to osteoporosis. The diagnostic abilities of each model, represented by the AUC, are presented in Table 4. The AUC in the female group all exceeded 0.75, indicating good diagnostic power, which was superior to that observed in the male group. In the women’s group, a 1-unit increase in AVI, BMI, and VAI decreased the risk of osteoporosis by 12%, 13%, and 17%, respectively. In the men’s group, AVI and BMI exhibited the most protective influence on bone health (OR = 0.82 and 0.80, respectively) among the 7 indices. The AUC derived from the ROC curve exceeded 0.75 in all the models for the women’s group.

**Table 4:** Multivariable logistic regression between anthropometric indices and osteoporosis

	Females				
	Crude model		Adjusted model		
	OR^c^ (95% CI)	*p* value	OR^c^ (95% CI)	*p* value	AUC^d^
WC	0.97 (0.96–0.99)	< 0.001*	0.96 (0.94–0.98)^a^	< 0.001*	0.77
HC	0.94 (0.92–0.96)	< 0.001*	0.93 (0.91–0.96)^a^	< 0.001*	0.77
AVI	0.91 (0.87–0.96)	< 0.001*	0.88 (0.82–0.93)^a^	< 0.001*	0.77
BMI	0.90 (0.86–0.94)	< 0.001*	0.87 (0.83–0.92)^a^	< 0.001*	0.77
VAI	0.84 (0.75–0.94)	0.003*	0.83 (0.73–0.94)^b^	0.004*	0.75
WHR	0.99 (0.97–1.01)	0.293	0.98 (0.96–1.01)^a^	0.139	0.75
WHtR	0.98 (0.96–1.003)	0.088	0.96 (0.93–0.99)^a^	0.004*	0.76

	Males				
	Crude model		Adjusted model		
	OR^c^ (95% CI)	*p* value	OR^c^ (95% CI)	*p* value	AUC^d^
WC	0.93 (0.92–0.95)	< 0.001*	0.93 (0.92–0.95)^a^	< 0.001*	0.70
HC	0.88 (0.86–0.91)	< 0.001*	0.89 (0.86–0.92)^a^	< 0.001*	0.71
AVI	0.82 (0.77–0.87)	< 0.001*	0.82 (0.77–0.87)^a^	< 0.001*	0.70
BMI	0.79 (0.75–0.83)	< 0.001*	0.80 (0.75–0.85)^a^	< 0.001*	0.72
VAI	0.99 (0.93–1.06)	0.775	1.01 (0.96–1.07)^b^	0.633	0.65
WHR	0.96 (0.94–0.99)	0.002*	0.96 (0.93–0.99)^a^	0.003*	0.66
WHtR	0.92 (0.90–0.95)	< 0.001*	0.92 (0.89–0.95)^a^	< 0.001*	0.68

OR, odds ratio; CI, confidence interval; WC, waist circumference; HC, hip circumference; BMI, body mass index; AVI, abdominal volume index; VAI, visceral adiposity index; WHR, waist-to-hip ratio; WHtR, waist-to-height ratio; AUC, area under curve

*Statistical significance with a *p* value < 0.05

^a^Adjusted for age, high-density lipoprotein, low-density lipoprotein, triglycerides, uric acid, calcium, albumin, and smoking and alcohol consumption

^b^Adjusted for age, low-density lipoprotein, uric acid, calcium, albumin, and smoking and alcohol consumption

^c^For OR values below 1, an increase in the adiposity index indicates a decreased risk of osteoporosis

^d^AUC represents the diagnostic accuracy of a model, where a value of 0.7 ≤ AUC < 0.8 suggests the model exhibits good diagnostic ability

### Nonlinear Models Between Adiposity Indices and Osteoporosis

**Fig. 2:**
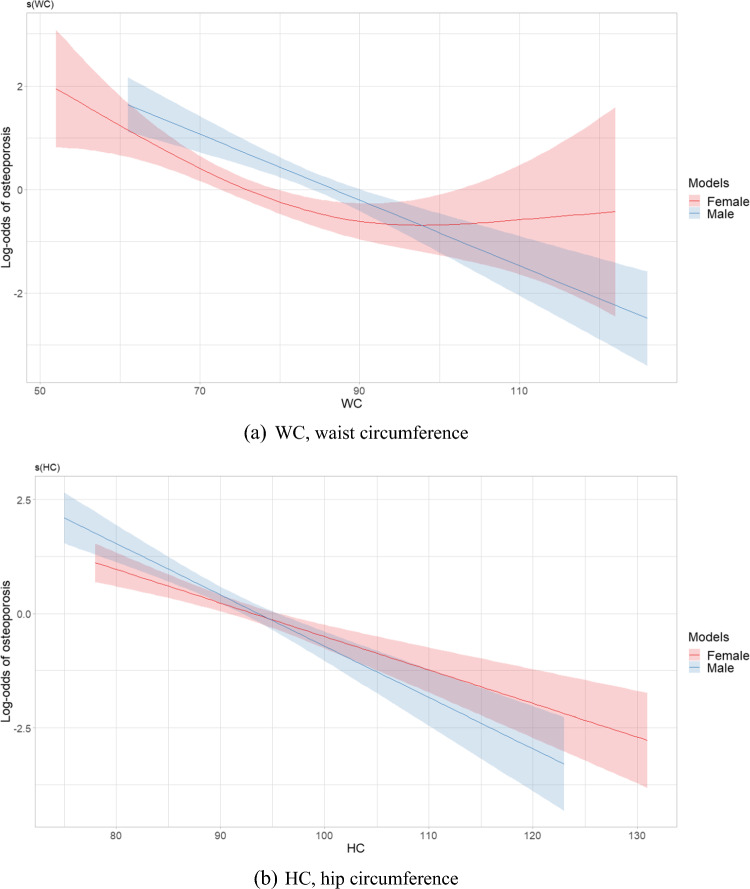

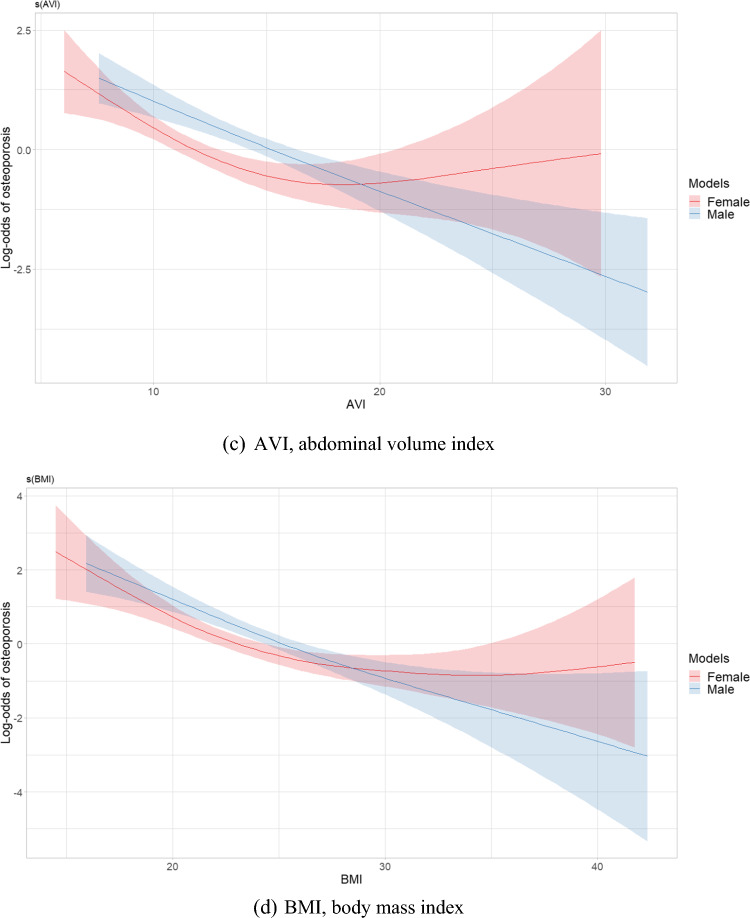

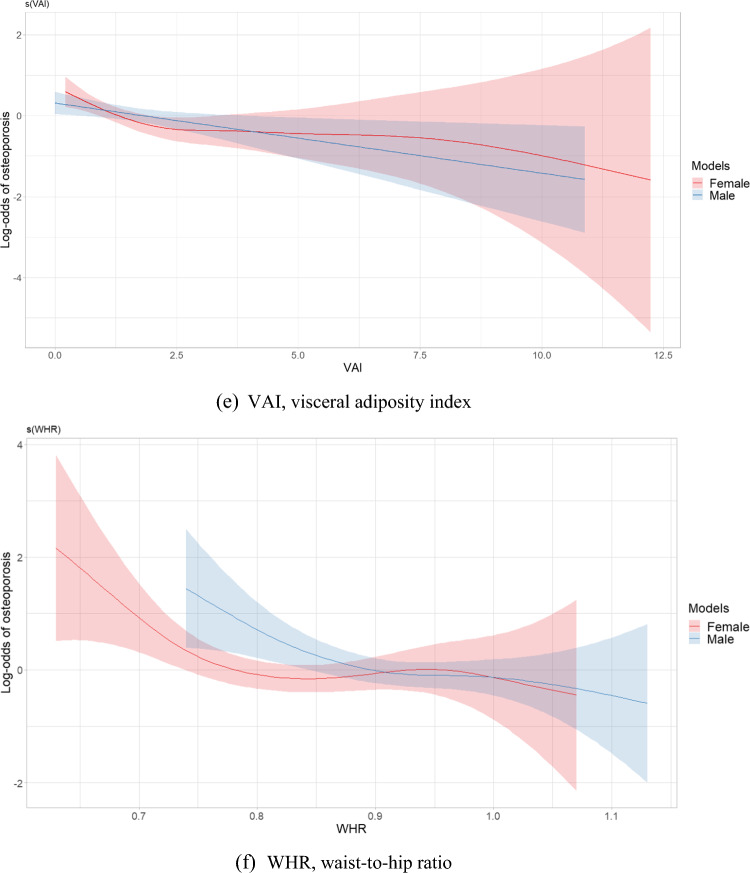

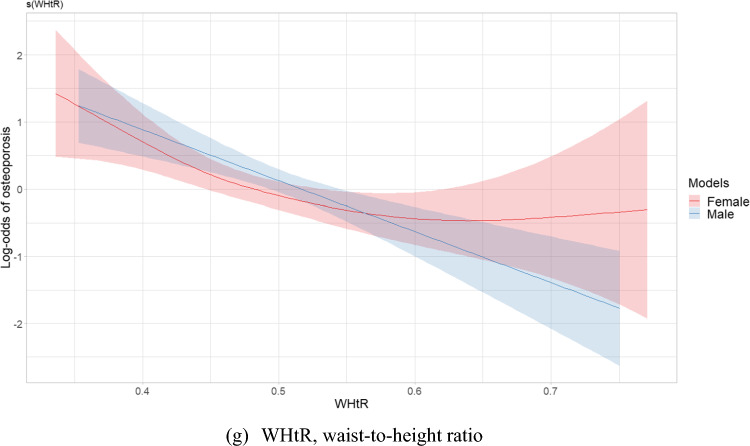
Estimated smoothing relationships of osteoporosis and obesity indices **a** WC (EDF = 2.49, *p* < 0.001 females; EDF = 1.00, *p* < 0.001 males), **b** HC (EDF = 1.001, *p* < 0.001 females; EDF = 1.00, *p* < 0.001 males), **c** AVI (EDF = 2.71, *p* < 0.001 females; EDF = 1.15, *p* < 0.001 males), **d** BMI (EDF = 2.81, *p* < 0.001 females; EDF = 1.52, *p* < 0.001 males), **e** VAI (EDF = 2.91, *p* = 0.006 females; EDF = 1.00, *p* = 0.012 males), **f** WHR (EDF = 3.21, *p* = 0.005 females; EDF = 2.25, *p* = 0.028 males), **g** WHtR (EDF = 2.39, *p* = 0.004 females; EDF = 1.00, *p* < 0.001 males) with adjustment for confounders using generalized additive models in postmenopausal women and men aged over 50 years. The x-axis refers to the value of explanatory variable and the y-axis refers to the log-odds of osteoporosis by generalized additive model. The shaded areas indicate the 95% confidence intervals. EDF, effective degree of freedom

Figure 2 depicts plots of smooth relationships between adiposity indices and the outcome for osteoporosis. Significant nonlinear associations were found for all indices in the women’s group, including WC (EDF = 2.49, *p* < 0.001), HC (EDF 1.001, *p* < 0.001), AVI (EDF 2.71, *p* < 0.001), BMI (EDF = 2.81, *p* < 0.001), VAI (EDF = 2.91, *p* = 0.006), WHR (EDF = 3.21, *p* = 0.005), and WHtR (EDF = 2.39, *p* = 0.004). An EDF value equal to 1 indicates a linear relationship, while an EDF greater than 1 suggests a nonlinear relationship and fits a more complex curve than a quadratic. The results revealed nonlinear protective effects of WC, AVI, BMI, VAI and WHtR. However, HC had a nearly linear association due to a low EDF value. U-shaped patterns of relationships with osteoporosis were identified in WC, BMI, AVI and WHtR**,** showing an initial decrease in the risk of osteoporosis as adiposity indices increased, followed by a reversal in this relationship after reaching a certain threshold. Conversely, a sinusoidal pattern of influence on osteoporosis was observed in VAI and WHR, reflecting a curve with alternating lows and highs, similar to a sine wave.

In the men’s group, specific indices, including AVI (EDF = 1.15, *p* < 0.001), BMI (EDF = 1.52, *p* < 0.001), and WHR (EDF = 2.52, *p* = 0.028), displayed nonlinear associations. However, AVI and BMI had a close to linear relationship with osteoporosis due to lower EDF values. In older men, indices such as WC (EDF = 1.00, *p* < 0.001), HC (EDF = 1.00, *p* < 0.001), WHtR (EDF = 1.00, *p* < 0.001), and VAI (EDF = 1.00, *p* = 0.012) exhibited a linear relationship with osteoporosis.

**Fig. 3:**
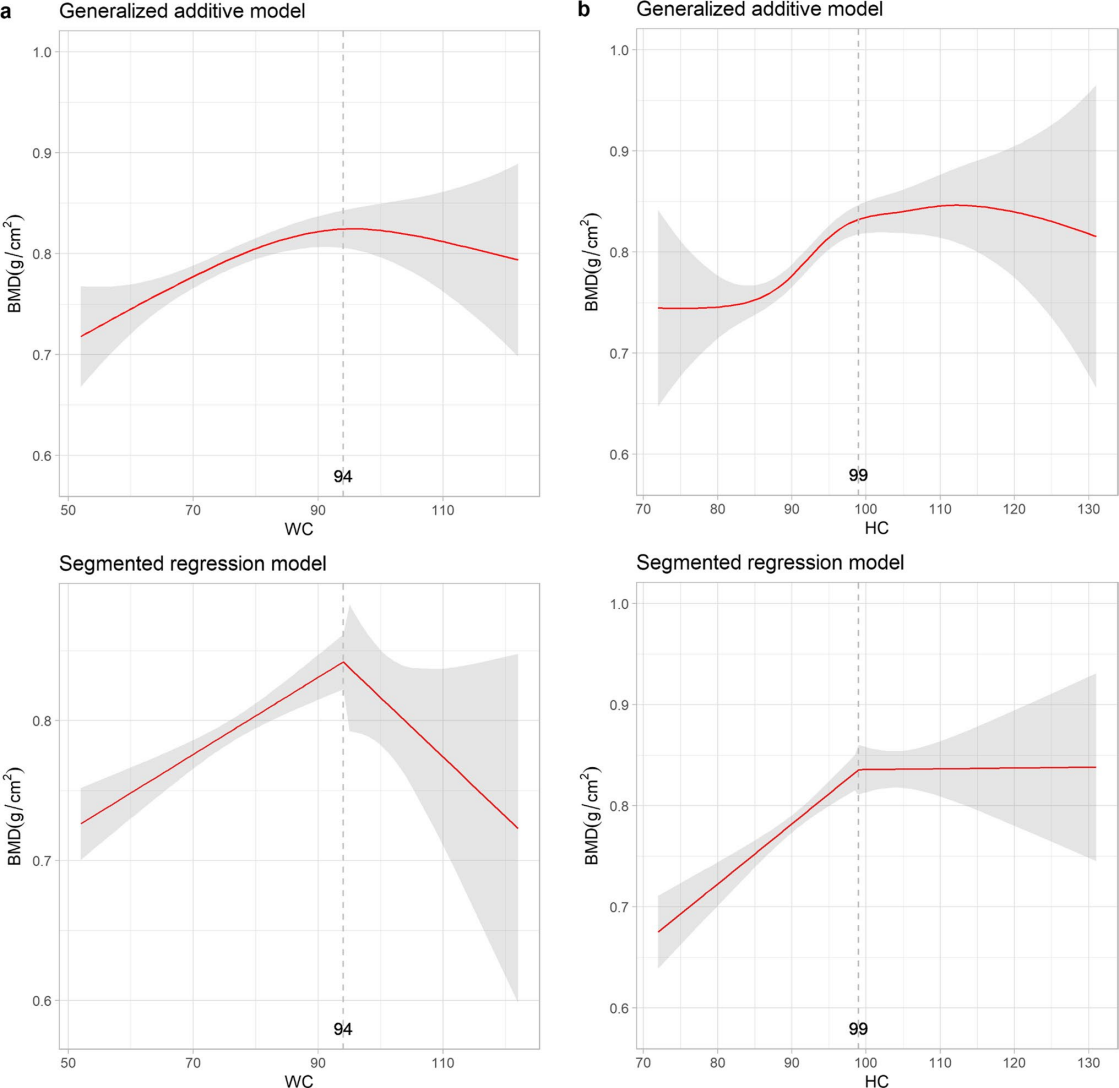

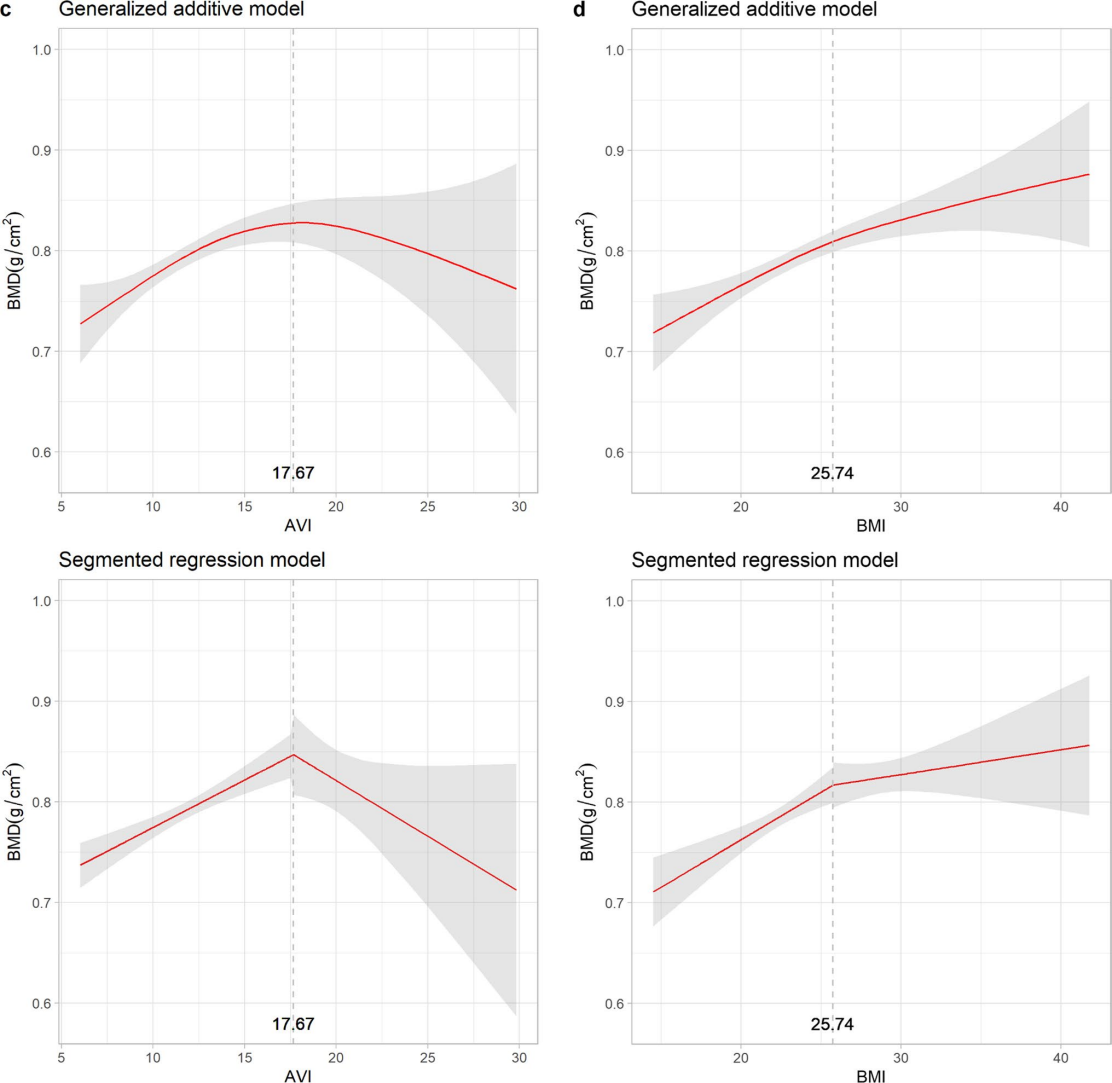

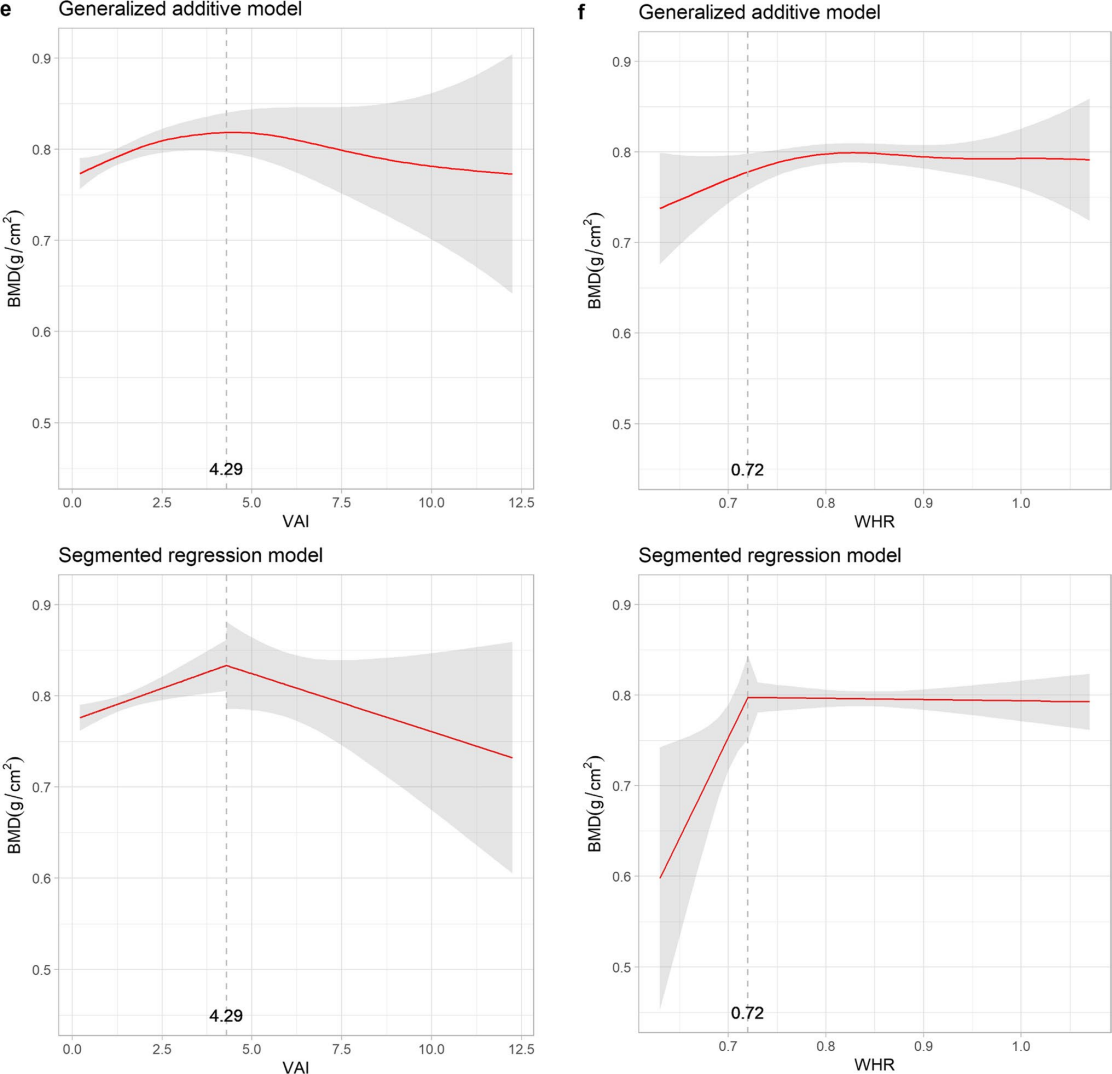

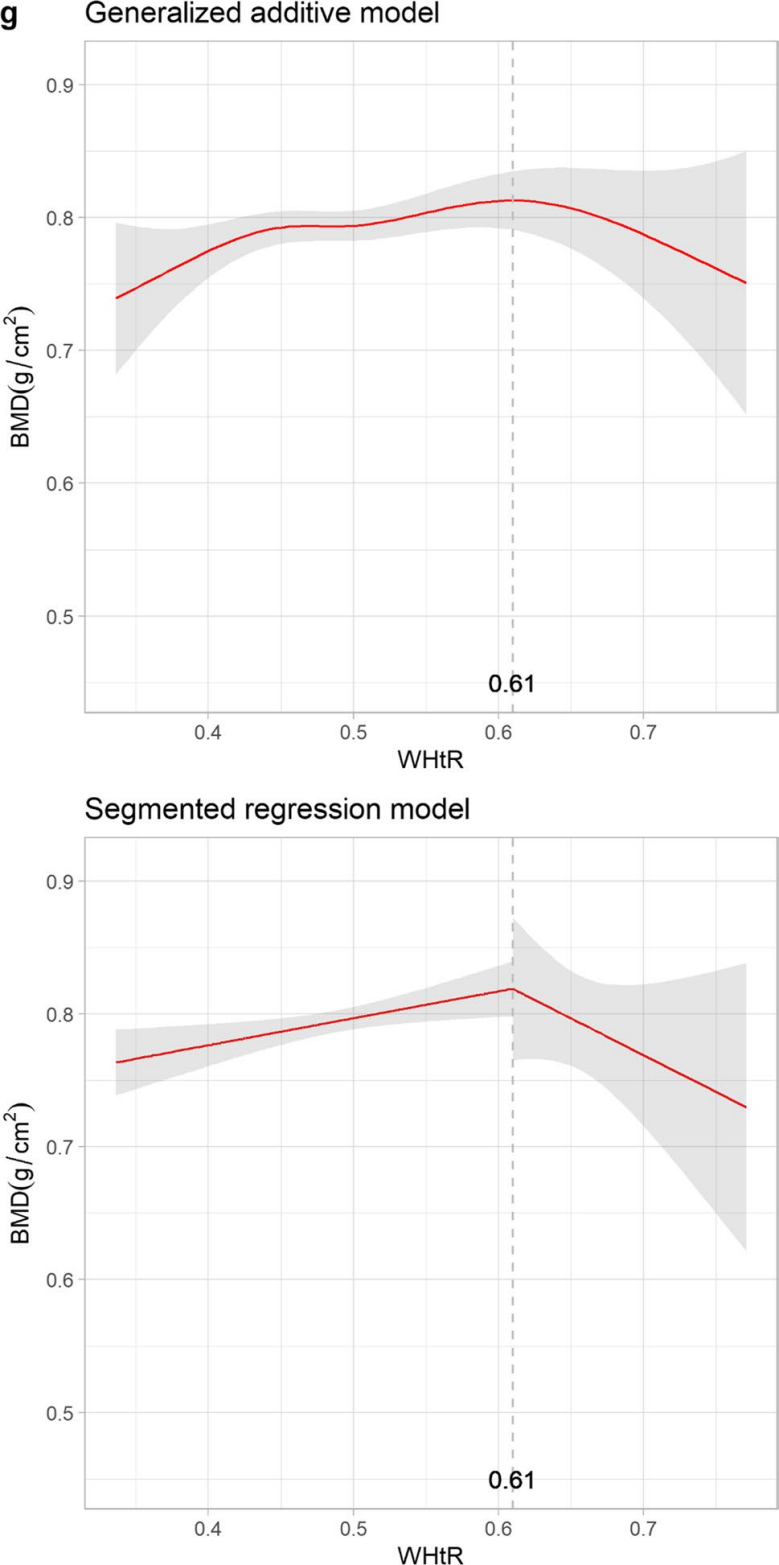
Relationships between bone mineral density and adiposity indices with thresholds: generalized additive model versus segmented regression model in postmenopausal women. The shaded areas indicate the 95% confidence intervals. BMD, bone mineral density; WC, waist circumference; HC, hip circumference; AVI, abdominal volume index; BMI, body mass index; VAI, visceral adiposity index; WHR, waist-to-hip ratio; WHtR, waist-to-height ratio

Given the U-shaped and sinusoidal patterns with potential inflection points observed in the relationships between osteoporosis and adiposity indices in postmenopausal women, Fig. 3 depicts the evaluation of BMD and adiposity indices using both GAM and SRM. Abrupt changes in BMD were observed in WC, AVI, VAI, and WHtR, with thresholds of 94 cm, 17.67 cm^2^, 4.29 and 0.61, respectively. Changes in BMD were less significant when exceeding an HC of 99 cm or WHR of 0.72. The magnitude of the positive association between BMI and BMD was less significant after 25.74 kg/m^2^.

## Discussion

To our knowledge, few studies have investigated the association between 7 distinct anthropometric measurements concerning the risk of osteoporosis, with a particular emphasis on the novel adiposity indices of AVI and VAI. Both AVI and VAI can be calculated during routine health examinations, offering valuable insights into the metabolic status of abdominal fat volume and visceral fat distribution in the study population. Early studies [29, 32, 33] primarily discussed the impact of traditional indices such as BMI, WC, and BW on osteoporosis, whereas recent interest has grown regarding the connection between AVI and bone health [28]. This study, unique in its approach and following the WHO and AACE osteoporosis diagnosis guidelines, incorporated conventional adiposity indices and novel adiposity indices, including AVI. Additionally, this represents a pioneering attempt to assess both linear and nonlinear associations between adiposity indices and osteoporosis. Our study differs from previous research by assessing the continuous interaction between adiposity indices and osteoporosis. This unique approach helps explain previous paradoxical findings [13, 14] and establishes a benchmark for optimal adiposity index levels to enhance BMD management and prevent the progression of osteoporosis. These results are particularly significant for postmenopausal women, providing a valuable reference for improving osteoporosis prevention strategies.

The major findings were as follows: First, the indices of WC, HC, AVI, BMI, VAI, WHR, and WHtR had nonlinear protective effects against osteoporosis in women with HC, exhibiting a nearly linear relationship with EDF = 1.001. We identified nonlinear patterns in both the GAM and SRM, which indicated an optimal body shape for postmenopausal women that avoids both unlimited weight gain or loss, thus aiding in osteoporosis prevention. We observed a threshold for protection against BMD loss associated with adiposity at WC = 94 cm, AVI = 17.67 cm^2^, VAI = 4.29 and WHtR = 0.61. A previous study reported that the average height of Taiwanese postmenopausal women is approximately 155 cm [34]. Our study results, combined WHtR of 0.61 with an average height of 155 cm, suggested a WC of 94 cm as a breakpoint, which aligns with the established WC threshold of 94 cm from the SRM result. Previous studies have shown that mechanical loading, estrogen conversion in fat mass, and insulin release support BMD, while cytokine secretion and vitamin sequestration inhibit it [15]. This interplay underscores the dynamic association through which obesity affects BMD and is consistent with our nonlinear findings. Second, in men, no apparent inflection point was found in the GAM, which serves as a linear indicator of bone mineral density deterioration. Third, despite the observed inverse linear correlation between VAI and osteoporosis in the GAM model for men, statistical significance was not achieved in the adjusted logistic regression. This discrepancy might due to influence of the fluctuations in TG levels, including variations depending on the day of the week when TG was measured [35]. These results suggest distinct impact patterns of optimal adiposity on osteoporosis in women and men, revealing nonlinear effects, particularly in women. The rapid decline in estrogen levels among postmenopausal women, compared to the gradual decrease in testosterone levels in aging men, may lead to less bone protection in women [36]. Additionally, men typically achieve higher peak bone mass and experience increased periosteal apposition during aging, contributing to greater net bone loss in women [37]. These differences likely play a role in explaining variations observed in the relationship between adiposity indices and osteoporosis across genders.

A key finding of this study was the significant role of AVI derived from anthropometric measurements of WC and HC as a valuable tool for assessing the association between adiposity and osteoporosis in both sexes. AVI was developed based on the concept of perceiving individuals as having conical or cylindrical shapes [19]. Previous evidence suggests that obesity-induced excessive fat accumulation could trigger the secretion of hormones such as leptin [38], adiponectin [39], and adipocytic estrogens [39], leading to the inhibition of osteoclastogenesis, increased bone formation, and higher bone mineral density [40–42]. In our study, a consistent trend was observed in which osteoporosis risk decreased with increasing AVI. However, the analysis also revealed a concave U-shaped impact pattern of AVI on skeletal protection for postmenopausal women, as demonstrated by the GAM and SRM analyses. Notably, an AVI ≤ 17.67 cm^2^ emerged as a critical indicator for the beneficial effects in gaining BMD and preventing osteoporosis.

To the best of our knowledge, this was the first study to investigate a non-linear negative association between AVI and osteoporosis. Prior research has primarily focused on the linear relationship between AVI and metabolic syndrome in both adolescents and adults [43, 44]. For instance, Wung et al. [28] identified a significant positive association between the AVI and T scores at the lumbar spine, femoral neck, and total hip in patients with metabolic syndrome, with an average AVI of 17.1 cm^2^. Our findings corroborate their results, indicating that an AVI ≤ 17.67 cm^2^ promotes BMD and reduces the risk of osteoporosis. However, their study included patients undergoing hemodialysis, which could potentially interfere with calcium metabolism. In contrast, our study, which was based on participants who underwent regular health examinations, offers findings with higher generalizability and is more suitable for evaluating the general population.

VAI, another contemporary adiposity index similar to AVI, targets abdominal obesity with a focus on visceral fat by incorporating WC, BMI, HDL, and TG into its calculation formula [20]. Previous research has demonstrated an inverse association between visceral fat tissue and BMD in men and women aged 30–50 years [45]. This observation could be explained by the negative association between visceral adipose tissue and bone markers such as carboxy-terminal crosslinked telopeptide of type 1 collagen and osteocalcin, suggesting that a higher VAI correlated with lower bone resorption and reduced bone turnover [46]. However, a sinusoidal-like decrease in osteoporosis risk associated with the VAI was noted in the women’s group, whereas no such pattern was observed in the men’s group. This difference could be linked to changes in body composition during menopausal transition in women, where both visceral adiposity tissue and total body fat increase [47]. Our findings are consistent with those of Sun et al., who used data from the U.S. National Health and Nutrition Examination Survey [48]. However, while their research primarily involved non-Hispanic White and Black participants, we provide results specific to an Asian population and demonstrate a universal change across different races. Our study suggests that a VAI ≤ 4.29 served as the threshold for protection from BMD loss and subsequent osteoporosis. This emphasizes the importance of considering the VAI as a significant adiposity index for assessing adiposity and osteoporosis in postmenopausal women. However, the nonlinear nature of this relationship must be acknowledged when interpreting the results.

Traditional adiposity indicators in our study including WC, HC, and BMI, significantly reduced the risk of osteoporosis in the multiple logistic regression models for both sexes. This is consistent with previous studies that reported similar outcomes for WC [14] and BMI [49–51]. Furthermore, these findings support the hypothesis that mechanical loading aids bone formation and hinders bone resorption [52]. In our GAM analysis of women, we noted a concave nonlinear relationship between bone destruction and WC and BMI indices. HC revealed a relationship close to linearity, with an EDF of 1.001, consistent with the logistic regression. According to the SRM results, maintaining WC ≤ 94 cm was associated with favorable protective benefits, and the positive effects on BMD from BMI diminished after reaching a BMI of 25.74 kg/m^2^. However, there was a lack of consistency in the relationship between WHR and osteoporosis as well as between WHtR and osteoporosis. Previous studies have primarily recognized WHR as a risk factor for osteoporosis [51, 53]. However, Tian et al. [54] proposed a contrasting view, suggesting a protective effect of both WHR and WHtR against bone loss. In our study, a nonlinear association between WHR and osteoporosis was identified using the GAM analysis for both sexes. The observed sinusoidal relationship may help clarify the inconsistencies observed in previous studies. Specifically, in the SRM analysis of WHR for women, the BMD reached a plateau after WHR = 0.72. Our findings provide clinicians with valuable reference data for assessing BMD in postmenopausal women. They underscore the importance of early intervention strategies, such as dietary modifications to include adequate calcium and vitamin D intake, as well as weight-bearing exercises, when these indicators exceed their threshold levels. However, discrepancies in WHR may be attributed to variations in the measurement methodologies. We measured WC and HC according to the WHO expert consultation guidelines [18]. The protocol defined WC as measured using palpable landmarks, specifically the midpoint between the lower margin of the rib and the top of the iliac crest. HC was measured at the widest part of the buttocks. Our measurements strictly followed the WHO protocol and were performed by trained nurses to minimize potential bias and possible observer and technique errors.

We found a linear correlation between bone structure deterioration and traditional adiposity indicators, such as WC, HC, and WHtR in men participants, providing a straightforward estimation of skeletal health in male patients in clinical settings. Therefore, indicators such as WC, HC, and WHtR served as simple tools for assessing osteoporotic risk reduction in men, with 7%, 11%, and 8% drops in osteoporosis risk per increment in WC, HC, and WHtR, respectively. Notably, the models incorporating HC in multivariable logistic regression demonstrated the highest diagnostic accuracy, with an AUC of 0.71. This highlights the significance of the readily accessible HC measurement in evaluating osteoporosis risk in men within a clinical setting. BMI had an inverse effect on the risk of osteoporosis, exhibiting a nonlinear pattern in both the men’s and women’s groups, with no obvious inflection points for men. Caution is advised when applying WHR in osteoporotic assessment because of its sinusoidal pattern of impact. The accumulation of adipose tissue promotes pro-inflammatory cytokines and enhances osteoclast differentiation and the resorption process [55]. Therefore, for postmenopausal women aiming to preserve BMD and prevent osteoporosis, maintaining a slightly overweight body shape with a BMI < 25.74 kg/m^2^, WC < 94 cm, or WHtR < 0.61 is beneficial.

The current study has several limitations. First, the dataset lacked information on exercise habits. However, querying exercise habits could potentially introduce recall bias. Additionally, all participants voluntarily enrolled in our prospective study without being referred for further evaluation of osteoporosis or obesity. Therefore, exercise was not considered as a covariate in our analysis. Second, the cross-sectional design prevented causal inferences. However, osteoporosis diagnosis was made by DXA based on the gold standard from the WHO, and we included almost all possible confounders, including personal habits, calcium levels, and anthropometric measurements without collinearity. Further longitudinal studies that include hormonal changes in both genders and strictly control the timing of TG measurements may provide more evidence regarding gender differences in the effects of adiposity indices on osteoporosis. This approach could reduce potential biases from TG fluctuations and improve VAI calculations.

## Conclusion

In conclusion, our study indicates that adiposity has different patterns of impact on osteoporosis in postmenopausal women and men aged over 50 years. In women, adiposity effects displayed a nonlinear concave pattern, with sinusoidal wave patterns observed in VAI and WHR. To maintain BMD and reduce the risk of osteoporosis, we recommend adhering to the following thresholds: WC = 94 cm, AVI = 17.67 cm^2^, BMI = 25.74 kg/m^2^, VAI = 4.29 or WHtR = 0.61, along with support from weight-bearing exercise and dietary calcium supplement. In men, while WHR showed a nonlinear sinusoidal relationship with osteoporosis, AVI, BMI, WC, HC, and WHtR were identified as linear protective factors. The gender difference observed may be attributed to hormonal variations and changes in periosteal dynamics during aging. These findings highlight the importance of using specific adiposity indices to assess osteoporosis risk differently in men and women, particularly providing insight into the continuous U-shaped interaction of adiposity and osteoporosis in postmenopausal women. Utilizing readily accessible anthropometric measurements enhances clinical decision-making or follow-up practices, and informs the development of public health policies. Future research should focus on confirming these results and exploring the underlying mechanisms, including biological and hormonal changes, the timing of TG measurements and the interaction with exercise, using longitudinal approaches. Understanding these relationships better can enhance clinical strategies for osteoporosis prevention and management.

## Data Availability

All data generated during this study are accessible from the corresponding author upon reasonable request in accordance with the hospital's research regulations.
